# Supporting the nurse educator in clinical education – a qualitative evaluation of a digital educational resource DigiVIS

**DOI:** 10.1186/s12912-023-01599-w

**Published:** 2023-11-16

**Authors:** Kristin A Laugaland, Maria Handeland, Ingunn Aase, Anne Marie Lunde Husebø, Christina Frøiland, Kristin Akerjordet

**Affiliations:** 1 SHARE—Centre for Resilience in Healthcare, Faculty of Health Sciences, University of Stavanger, Kjell Arholms Gate 41, Stavanger, 4036 Norway; 2 Department of Care and Ethics, Faculty of Health Sciences, University of Stavanger, Stavanger, Norway; 3 Department of Public Health, University of Stavanger, Stavanger, Norway

**Keywords:** Clinical placement, Digital educational resource, Nursing, Nurse educators, Nursing homes, Qualitative

## Abstract

**Background:**

Despite the increased use of technology for teaching and learning in clinical nursing education, relatively little attention seems to be directed toward the usefulness of digital educational resources (DERs) to support nurse educators’ educational role in clinical nursing education.

**Methods:**

An interpretive descriptive qualitative study design was conducted to evaluate the usefulness of a DER to support nurse educators in clinical nursing education. Data were collected through two focus group interviews with part-time and novice educators (*n* = 5) and full-time, more experienced educators (*n* = 5), after they had overseen student nurses in nursing home placements. Data were analyzed using thematic analysis and Standards for Reporting Qualitative Research guidelines were used for this study.

**Findings:**

The analysis identified three themes related to nurse educators’ experiences of the usefulness of a DER to support their educational role while overseeing first-year students on clinical placements in nursing homes: (1) Provides academic support and a sense of security (2) promotes pedagogical efficacy, and (3) represents a flexible resource for educational planning.

**Conclusion:**

This study shows that a digital educational resource can be an efficient and useful supplementary strategy to support the nurse educator’s role in clinical nursing education. Future research is required to systematize knowledge about the impact of DERs on orientation and training, as well as motivation and facilitators for, and barriers to, their use to enhance quality and strengthen the nurse educator’s role in clinical nursing education.

**Supplementary Information:**

The online version contains supplementary material available at 10.1186/s12912-023-01599-w.

## Background

 For clinical placements in nursing, the role and competencies of the nurse educator are important to enhance student nurses’ learning and professional development, thus supporting the education of innovative future nurses [1, 2]. Student nurses perceive teaching and supervision by nurse educators in clinical placements as a key part of learning evaluation, integrating theoretical and practical knowledge, understanding the student’s role, and providing emotional support [3]. Consequently, teaching effectiveness should be demonstrated by an adept personal teaching style, the use of different teaching strategies, and expert knowledge of the subject [4]. Surprisingly, no consensus exists however regarding the minimum qualifications or required competencies for nurse educator’s role in clinical education across institutions and countries [5–7]. Nonetheless, there appears to be agreement that nurse educators must have expertise, as previously stressed, within the practice of nursing as well as in education [8].

The nurse educator’s role in clinical placements varies depending on which clinical placement model is applied. In this study, the term “role” indicates the sum of expectations and norms linked to the specific position and function of a nurse educator in clinical nursing education. The focus of the current study, in a Norwegian context, like other countries in Europe most often uses the preceptorship model in nursing education [9]. In this model, the students are most often allocated to a registered nurse (RN) employed by the clinical placement site who take on the preceptor role and has the overall day-to-day mentoring responsibility as part of their authorization. In Norway, RNs do not receive financial compensation for being a preceptor, and no formal preceptorship requirements exist. This implies that preparation for the preceptor role varies across nursing programs. Nevertheless, within the preceptorship model the nurse educator is responsible for coordinating nursing student learning, supporting students’ integration of theory with practical learning, and on students achieving their learning outcomes considered as a liaison role [9]. The nurse educator, who is employed by the higher educational institution (HEI), has therefore a more advisory, collaborative, and supportive role in relation to the RN preceptors requiring high degree of nursing and pedagogical competence [10]. Nurse educators’ support is emphasized as pivotal in nursing home placement settings given the shortage of RN preceptors available to support student nurses learning in this context [11].

Nurse shortage is a global challenge also affecting the educational sector [12]. Part-time hired nurse educators are therefore commonly used for clinical education in nursing homes due to a shortage of available nurse faculty staff [11, 13]. As clinical practice education constitutes 50% of the bachelor education program in Norway and elsewhere in Europe, it is a resource-intensive aspect of nursing education. It has been necessary to recruit nurse educators on temporary contracts to ensure that the increased intake of student nurses has sufficient support from nurse educators during clinical placements [11]. Nurse educators hired part-time or on temporary contracts are often experienced clinical RNs hired to take on an educational role as nurse educators during students’ clinical placements. However, it is widely acknowledged that the transition from a clinical practice nursing role to an education role is demanding, as teaching requires “a skill set of its own” [4]. This implies that nurse educators may struggle to facilitate optimal learning when supervising students in unfamiliar environments [14].

Concern and challenges related to a shortage of nursing faculty staff have been documented for decades [15]. In this regard, the World Health Organization [16] pinpoints that the quality of educational preparation of nursing faculty represents a growing concern. Nevertheless, guidelines to assist clinical and novice nurse educators on effectively teaching and supervising student nurses are lacking [17]. As a result, nurse educators face challenges and may not adequately teach, guide, supervise, and assess student nurses during clinical placements, a situation which may reduce their effectiveness as educators [4, 8]. These concerns give rise to a growing need for more innovative methods that allow a transformative pedagogical practice and innovative ideas and techniques to orient, mentor, and develop nurse educators [11, 18].

Initiatives to use digital educational technology for the teaching and supervision of students to complement learning are increasingly being adopted and considered of importance in nursing education to dynamize teaching and encourage future nurses to undertake active learning projects and acquire the necessary skills [11]. However, even though there has been an increase in the use of technology for teaching and learning in clinical nursing education [19], relatively little research attention appears to be directed towards the usefulness of digital educational resources (DERs) to support the nurse educator’s role in this area. This study addresses this knowledge gap by evaluating nurse educators’ perceptions and experiences of the usefulness of a DER (DigiVIS) to support their educational role in clinical nursing education.

## Methods

### Design and setting

An interpretive descriptive qualitative study design using focus group interviews was applied. This design was chosen as it ensures responsiveness to experience-based questions of interest to a practice-based discipline such as nursing [20]. According to Krueger and Casey [21], the focus group method creates a space for group interaction and discussions that are expected to yield rich and informative data. This assertion supports the idea that focus group interviews are beneficial for researchers since they provide insight into social relations, and the information obtained may better reflect overlapping knowledge than a summation of individual interviews [21].

The nurse educators who participated in this study came from an HEI in Norway. In Norway, student nurses undertake their first clinical placement in nursing homes. In our study, this was an eight-week mandatory clinical placement. The preceptorship model described above was applied at the HEI enrolled in the study. Standards for Reporting Qualitative Research guidelines were used for this study [22].

### Recruitment and participants

Recruitment of nurse educators was based on a purposive criterion-based sampling strategy [23]. The strategy was chosen to make sure that both experienced and novice nurse educators were included to strengthen the explorative nature of our research. We sought to recruit full-time, more experienced nurse educators as well as part-time or newly employed ones, including those on a temporary contract, to represent novice educators. The sampling strategy employed in this study was thus considered a reasonable approach that would allow the researchers to benefit from the nurse educators’ diverse viewpoints, experiences, and understandings [e.g., 23]. Prior approval from the Vice-Dean of Education at the enrolled HEI was attained. When potential participants were identified, an email invitation with a written consent form and information about the study was sent to eligible nurse educators. A total of six part-time or novice educators and four full-time educators participated in this study (*n* = 10). All participants gave written consent.

The enrolled nurse educators ranged in age from their early thirties to over sixty with an average age of 43. All were women. They spent between 20% and 100% of their working hours at the HEI in question. Two of the participants were on temporary contracts. Four of the ten participants had no or little (> 1 year) prior experience as a nurse educator in clinical education, while six had from five to 17 years of experience. Only three of the educators had formal pedagogical education. One of the experienced nurse educators had further education in geriatrics (i.e., a Master of Science degree in healthcare services for the elderly). During the student nurses’ clinical placement in nursing homes, each nurse educator was responsible for between two and 14 students with an average of seven. Table 1 summarizes the key characteristics of the study participants and the focus groups to which they belonged.

**Table 1:** Participant characteristics and focus groups to which they belonged

Participant	Age	Employment	Experience as nurse educator in clinical nursing education	Number of students during this clinical placement period	Formal educational training: (yes/no)	Further education in geriatrics: (yes/no)
Focus group interview A: Part-time or novice educators.						
#P1	50	Temporary contract	Novice	2	no	no
#P2	33	New full-time educator		7	no	no
#P3	32	Part-time educator	Novice	5	no	no
#P4	55	Part-time (20% position)	11 years of experience	11	yes	no
# P5	36	Temporary contract	Novice	5	no	no
Focus group interview B: Full-time and experienced educators.						
#P6	47	Full-time	10 years of experience	14	yes	no
#P7	60	Full-time	17 years of experience	8	Yes	no
#P8	44	Full-time	6 years of experience	12	no	no
#P9^a^	65	Part-time (20% position)^a^	6 years of experience	5	no	yes
#P10	54	Full-time	9 years of experience	6	yes	no

^a^Participant #P9 was not able to attend focus group interview A when scheduled and was thus included in focus group B even though she worked part-time. \

### Orientation to prepare part-time and novice nurse educators for the educational role in clinical education at the enrolled HEI

At the HEI enrolled in the study, the standard preparation and orientation program offered to part-time and novice nurse educators entails four physical orientation meetings before a placement combined with two physical meetings arranged by the course director during the placement period (i.e., prior to the midterm and final assessments) to offer support and guidance. Topics covered during orientation include an overview of policies and procedures and a description of the roles and responsibilities of a nurse educator in clinical education. As this study was carried out during the Covid-19 pandemic, the orientation course had to be arranged and held digitally by the clinical course director to prepare participants for their educational role when overseeing students in nursing home placements, including supervision and assessment practices. Beyond this, the clinical course director also sent paper files to all the nurse educators via email before the placement period with the course description, describing the students’ learning objectives, assessment forms, and other practicalities. All the educators were offered access to the digital learning platform used at the HEI, where they could see the students’ preplacement orientation and teaching. The course director also arranged for and invited the novice and part-time educators to follow up with digital meetings during the placement period to offer support and guidance before their assessment discussions and for dialogue and to answer questions when needed. The orientation program and follow-up meetings are not mandatory for part-time or novice educators at the enrolled HEI. Participation in the orientation and follow-up meetings was, however, not reported in the study.

### Description of the DER being evaluated in the study

As part of a larger study [24], the DigiViS (DER) being evaluated in the study was co-created with key stakeholders (i.e., student nurses, nurse educators, RN preceptors, e-learning designers, and researchers) and originally designed to enhance RNs’ preceptorship practices of student nurses in nursing home placements [25]. For a more detailed description of the co-creative process, see Laugaland et al. [26]. As part of, and in parallel to, pilot testing and evaluating the DigiViS resource with RN preceptors, we also invited nurse educators to test and evaluate the DER during the same time span to inform supporting interventions.

**Fig. 1:**
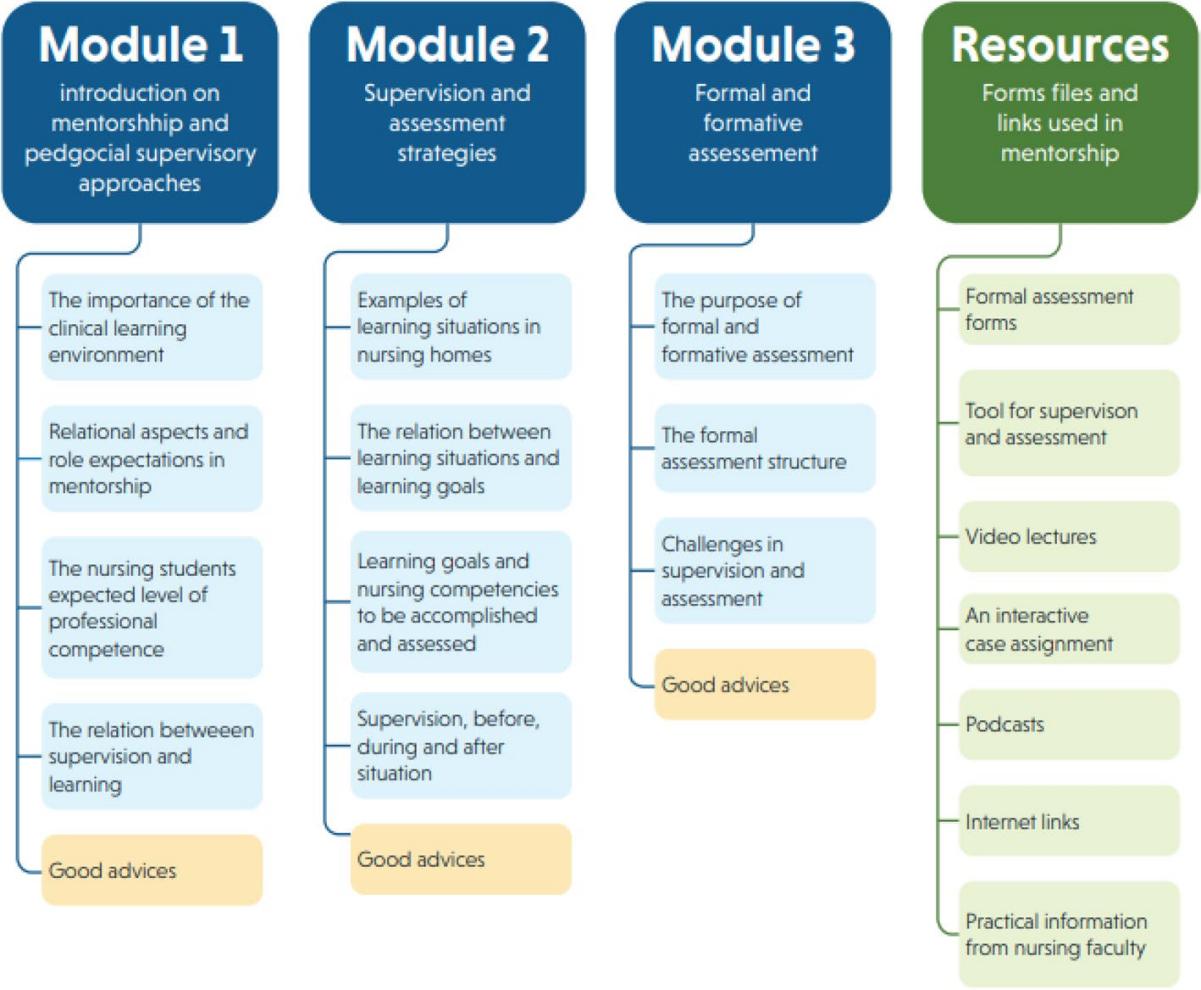
Content overview of DigiViS

The DigiViS resource was offered to the participants enrolled in the study via access to a web link one week before their placement period. The participants also had access to the resource throughout the placement period. The DigiViS consisted of three 15-minute modules (see Fig. 1) related to the pedagogical content knowledge of the clinical education program in nursing homes at the enrolled HEI. The three modules were organized with different topics and had the following titles: *Introduction to mentorship and pedagogical supervisory approaches; Supervision and assessment strategies;* and *Formal and formative assessments.*

The first module contained literature on pedagogical supervisory approaches that emphasized the importance of the clinical learning environment, the relational aspect in supervision, and the relationship between supervision and learning. It also included role expectations and an overview of the students’ educational context and thus their expected level of professional competence. The second module provided examples of learning situations tailored to accommodate the students’ learning objectives as well as a description of their competence areas. The last module focused on providing information about the purpose of formal and formative assessments in clinical education in nursing homes and describing the assessment forms used. In addition to theoretical knowledge, each module included illustrations, various digital resources, such as podcasts and video lectures, and reflective activities. All modules ended with advice and summaries of key module content.

### Data collection

Two semi-structured focus group interviews were conducted with the part-time and novice educators (i.e., focus group A [FGA]) and full-time or more experienced educators (i.e., focus group B [FGB]) following the students’ nursing home placement period in April 2021. Separate focus group interviews allowed the researchers to explore how DigiViS was experienced differently by part-time or novice educators and full-time, more experienced ones. Participants in the first focus group (i.e., FGA) were part-time, external nurse educators on temporary contracts and novice educators, while FGB comprised full-time and more experienced educators. Note that one part-time nurse educator (#P9 – see Table 1) participated in the focus group interview with the full-time educators as this participant was not able to attend the focus group interview scheduled with part-time and novice educators. This was deemed appropriate as this participant had six years of experience as a nurse educator in a part-time position at the enrolled HEI.

Due to the Covid-19 pandemic, online semi-structured focus groups were conducted via Zoom. The focus group interviews were conducted by experienced qualitative nurse academic researchers. The semi-structured interview guide addressed themes such as functionality, content, relevance, usefulness, and improvement. The interview guide permitted spontaneous reflections from the participants at the end of the interviews, which also included questions and reflections from the moderator. The interviews lasted approximately 60 min and was moderated by the first author while the third and fourth authors took field notes following up by clarifying questions.

### Ethics approval and consent to participate

The study was approved by the Norwegian Centre for Research Data in line with the European General Data Protection Regulation (GDPR) (2018/61309 and 489776). Participation was based on informed and voluntary written consent, and the participants had the right to withdraw at any time without any negative outcomes. They were also assured that the information would only be used for the purpose of this research project. All data were treated confidentially and stored carefully and securely in accordance with national regulations.

### Data analysis

Braun and Clarke’s [27] six-step model inspired the data analysis, which was an iterative process: First, the text was read several times by the first and last author to familiarize and to gain a sense of patterns of meaning. They then shared and discussed summaries of their overall impressions,  second, initial codes were generated by the first and last author based on the study aim, to determine patterns, third, recurring themes forming patterns were searched for in light of the coded data covering nurse educators’ perceptions of the usefulness of the DigiViS, forth, the initial themes were discussed and reviewed by the first and last author, fifth, all authors were engaged in discussions around defining and naming the themes and potential sub-themes through a consensus process, and finally, the report was produced. Examples of the themes (i.e., quotations) were compared across the data items (i.e., interviews and participants), and the most illustrative were selected for each theme.

### Findings

The analysis identified three themes with associated sub-themes, as illustrated in Table 2, related to nurse educators’ perceptions of the usefulness of DigiVIS to support their educational role while overseeing first-year students during their clinical placement in nursing homes: 1) Provides academic support and security, 2) promotes pedagogical efficacy, 3) represents a flexible resource for educational planning. These themes are described in more detail below.

**Table 2:** Overview of themes and associated sub-themes

Main themes	Sub-themes
Provides academic support and a sense of security	• Support in a new and unfamiliar role • Insight into the student’s educational context • Feeling more secure and supported • Confirming professional practices • Enhancing uniform practices
Promotes pedagogical efficacy	• Linking theory and practice • Supporting formal interactions and assessment discussions • Providing a shared ground • Promoting reflective thinking as a teaching strategy
Represents a flexible resource for educational planning	• Flexible • Easily accessible • Information can be accessed at your own pace • Educational preparation

### Theme 1: provides academic support and a sense of security

Even though DigiViS was originally developed to enhance RN preceptors’ supervision and assessment competencies, all the nurse educators were enthusiastic and positive about this DER. All the participants emphasized that DigiViS was transferable and useful in terms of providing academic support, such as strengthening professional insight into a student nurse’s educational context, including their current level of expected nursing competencies, and describing the achievement and assessment of a student’s learning goals. Moreover, DigiViS was described as useful in terms of providing a sense of security, confirming professional practices, enhancing training, and promoting quality assurance.

There was some variability, however, across the experienced and novice educators’ experiences of and reflections on the usefulness of DigiViS. The part-time and novice educators stressed that DER gave them valuable and necessary academic support that was very much needed for training purposes, as they expressed the need for adequate orientation, training, and support in a new and unfamiliar role as a nurse educator:

*You have practice, then you have this [education]. They are two different worlds, really. I felt that there were so many new things in the role as a nurse educator, yes, all the things and documents I have to deal with… I think with this resource [DigiViS]… yes, that my role as an educator is a bit more… I was about to say more reasoned, or that I can point to what professionalism actually is, that I can take care of the students better. So, I felt that this resource [DigiViS] was good for me. [#P1, FGA]*

All the part-time and novice educators emphasized that DigiViS provided them, in an easy and efficient manner, with valuable insight into the nursing students’ current level of nursing competence and their educational context as well as a description of the learning goals to be met by the students and assessed during the placement. One of the novice educators clarified why this knowledge was important:

*Now I can go out [on the placement] and stress that you [the students] should know this. And it is very good. Because we cannot know by heart… right. So, I think that it was very good that, in a way, it says it here. They have learned this, and it simply provides a summary of what they should learn, this they have had of theories… I think it [the content of DigiViS] covered a lot. It was good that it listed the students’ prior knowledge. Since I am not involved in education, I feel I knew little in advance of what the students should know or have had. [#P2 FGA]*

Moreover, several of the part-time and novice educators expressed that DigiVIS strengthened the academic part of their role and made them feel more secure and supported. Two said:

*So, this resource [DigiViS] contributed to the professional part, so I felt more secure about that. Yes, the competence goals, the extra professional weight, so I think that something would be missing if this [DigiViS] were not continued, so then I would lack something as an educator. [#P3 FGA]*

*Very good that we gained insight into the students’ prior knowledge. Because I felt I knew so little in advance about what the students really should be able to do or should have had or known. So, this resource [DigiViS] contributed to that professionalism*, *which gave me a sense of security when meeting with the students. [#P1 FGA]* 

The novice educators emphasized that without access to DigiViS they would have felt that they were exposed to their own shortcomings when approaching the students and the RN preceptors:

*I think that this resource[DigiViS] in a way… that I received this link and had it on my mobile has given me security. If not, I would have felt exposed. Yes, to give advice to the students, to answer the registered nurse mentors… it [DigiViS] provided me with valuable professional and academic support. As a new educator, you just get thrown straight out there. [#P3, FGA]*

The experienced nurse educators, on the other hand, expressed that the usefulness of DigiViS was more linked to how the resource confirmed and supported their existing professional practices but also provided advice and tips:

*The resource [DigiViS] gave me some good feelings in relation to a confirmation of things that I have done for many years… so it became a bit personal… confirming that I at least did something right in my role as an educator. [#P6, FGB]*

*I thought it [DigiViS] was very useful. So, you can go straight in and receive some good tips and advice – that you can take for granted when you have been in the game for several years – you do a lot of things on autopilot – so for me it was a good reminder, and you get a bit of confirmation of, yes, I am heading in more or less the right direction when it comes to this. I also thought this was good. [#P7, FGB]*

Furthermore, several of the more experienced nurse educators emphasized that a resource like this had been missing in clinical nursing education. One said:

*My first reaction to DigiViS was that I became wildly excited and knew that I had been lacking something like this for a long time. [#P6 FGB]*

When the experienced educators discussed and reflected upon the usefulness of DigiViS, several also emphasized that a professional, academic, and educational resource of this type could contribute to promoting quality assurance and spoke of the need to establish more systematic supervision in clinical nursing education. Several of the experienced educators said that a DER of this type could promote or ensure more consistency and common ground among educators and enhance uniform practices in student follow-up. One of the experienced nurse educators said:

*So, this [DigiVIS] kind of met a need to systematize supervision. And supervision cannot be uniform, true, because we are all different individuals who meet different student nurses, but with this [DigiViS], you have a common framework to guide us. [#P8 FGB]*

Another part-time experienced educator shared similar thoughts and reflections:

*Yes, I think that much of what is written here is directly transferable to us. This to learning situations, this to questions that stimulate reflection. Thus, the more we can talk about things in the same way, the better the result. [#P9 FGB]*

### Theme 2: promotes pedagogical efficacy

The second theme was that DigiViS, by providing academic support and a sense of security, promotes the pedagogical efficacy of the nurse educators’ role, including linking theory and practice, supporting formal interactions and assessment discussions, and promoting the use of reflective thinking as a teaching strategy. In this regard, the academic and educational content provided in DigiViS helped nurse educators to concretize learning outcomes, link learning outcomes more effectively to clinical learning situations, and build on theoretical knowledge. Both novice and experienced educators emphasized that the DER motivated them to identify and operationalize learning situations and theoretical aspects in practice in an expedient, concrete, and integrative manner providing a shared ground:

*Yes, it was useful to be able to see what the students have gained from teaching – and, in a way, to be able to build on that and help them bridge and integrate theoretical and practical knowledge more easily. [#P1 FGA]*

*Helping the students find good learning situations tailored to their learning goals is important. And I think that was good about the resource [DigiViS] – it gave you concrete examples of good learning situations linked to the students’ learning goals. It was the concrete part of the resource that I found useful. [#P8 FGB]*

Particularly the part-time and novice educators highlighted that the content related to the concretization of the learning outcomes and areas of nursing competencies that the students should pursue and achieve during the placement period was useful. They shared how this content helped them to better understand the student learning outcomes, which they emphasized was beneficial in supporting them in their formal interactions with both students and RN preceptors as well as in fostering feelings of confidence.

*I found that it was very useful, and then I understood more of the competence targets which was important for me and gave me a sense of security. [#P3 FGA]*

*This review of the areas of nursing competence was so concrete and straightforward, I think. Because it’s not always easy to get the RN mentors to understand these, they don’t always know what's meant by them… nor do the students, for their part. Because they haven’t always familiarized themselves with the documents and information they have been sent. So, this [DigiViS] helped me to translate these learning outcomes in a more concrete way in dialogue with the RN mentors and students. [#P5 FGA]*

This perception was supported by the experienced nurse educators, who emphasized that DigiViS made it more likely to provide shared ground when focusing on concrete learning situations linked to learning objectives or outcomes at the same time boosting their awareness and equality of the need for teaching to be carried out in a uniform manner.

*But it is that we become more alike… Because they [the students] often say that you [educators] are so different, and then we often have replied yes, but then you learn differently, and we have shared goals, shared learning outcomes, and we can do things differently, we have this pedagogical freedom. I, who have been going on autopilot, had an “aha!” experience. Oh yes, this is what I must remember. [#P8 FGB]*

Both the novice and the more experienced educators stressed that the pedagogical content of DigiViS also acted as a reminder and support to promote the students’ reflective thinking skills, which they experienced as beneficial for supervising, interacting with, and discussing assessments with students:

*It was so useful, with good questions for reflection. The fact that it can become so concrete, that there are suggestions for questions that stimulate reflection, because that is what we want. We don’t just want an answer, we want the students to reflect. [#P7 FGB]*

*I also used these questions for reflections, I thought they were good when preparing for formal meetings with the mentor or student. [#P5 FGA]*

The DER was further welcomed when the nurse educators prepared for their formal tripartite meetings, including assessment discussions. For example, DigiViS helped the part-time and novice educators to reformulate the assessment form, which was perceived as abstract, into more comprehensible language that they perceived as encouraging the RN preceptors and student nurses to become more actively engaged in the assessment dialogue.

*Now I must reformulate this [the assessment form] into something a little more understandable. And then there was a better response from the RN mentor and from the student. [#P5 FGA]*

Beyond this, DigiViS and the reflections on it seemed to increase the nurse educators’ motivation and inspiration to do the best possible job in clinical educational contexts:

*So, I am very motivated to do the best possible job now for these students, so they can come out to a clinical placement and be prepared to learn and do whatever ensures our patients receive the best possible care. [#P10 FGB]*

### Theme 3: represents a flexible resource for educational planning

Participants across the two focus group interviews emphasized the usefulness of DigiViS being a flexible resource for educational planning. Part-time, novice, and experienced educators all consistently expressed the usefulness within their educational role of having easy access to the DER as it allowed them to access information at their own pace. DigiViS seemed to provide novice educators with a valuable source for planning and preparation beyond the ordinary preplacement training offered to part-time and novice educators. During ordinary training, the part-time and novice educators experienced that too much information was conveyed at once, causing stress and a sense of being overwhelmed. The information was therefore hard to remember, which became a barrier to reaching out for help. Access to DigiViS was thus very much appreciated as it represented something they could keep at hand, enabling them to absorb content and information at their own pace and go back and forth as needed:

*There has been so much new in the role of nurse educator, all the documents, practical booklet, assessment forms. I’ve had enough of digesting everything I must deal with – there’s so much information – that I forget a lot of it. So, the fact that everything is brought together in this resource [DigiViS] has been great – something I can return to if necessary. [#P2 FGA]*

As the part-time and novice educators thus had access to information and resources, they did not necessarily have to remember them but could easily access and revise them when needed. The DER also appeared to support the nurse educators’ need to educate themselves when time was available to them. One emphasized that having easy access to resource materials such as a digital lecture on the nursing process gave her an opportunity to update herself on the importance of this topic in supervising student nurses.

*So, if I have a free moment somewhere, I can easily look it up [in DigiViS] and read… simply educate myself on what I should have at hand, so I’m prepared to help the students as much as possible when I am talking to them. Then it is easier for me to do things on the spur of the moment. [#P5 FGA*

Beyond this, participants of all levels of experience emphasized the flexibility of the DER and observed that its accessibility on mobile devices allows them to plan right up to their meetings with nurse students and RN preceptors. One of the participants said:

*I really liked the fact that you could open it [DigiViS] on the mobile, on my iPhone. This was very useful. I used it and checked like, in the car, for example before I had to go in [to meet the students of their preceptors], and it was so easy to access and find tips and advice when preparing for these meetings. [#P3 FGA]*

The nurse educators appeared to have individual preferences about how to access the various resources offered by DigiViS. Several participants emphasized the value of the different ways the information could be acquired, for example, podcasts, video lectures, written text, or all three, and that they could select the resources they found most suitable for them individually:

*I think it was very good with those podcasts, that it [DigiViS] was also read aloud, so you could choose to listen instead of reading. When I was walking the dog, I could listen to the content of a podcast and get it on my mobile. [#P4 FGA]*

The accessibility of the DER was also acknowledged from a more long-term perspective and even identified as significant for all key stakeholders related to the student nurses’ clinical placement in nursing homes:

*I do believe that nearly everyone would be positive about simple support material provided in a DER about how one can help students. I think that it will be a good support for all three parties actually [students, educators, and RN preceptors]. [#P10 FGB]*

## Discussion

The findings from this study suggest that a DER such as DigiViS can be a useful innovative teaching and learning resource and an efficient supplementary strategy to support and enhance the nurse educator’s role in clinical nursing education. The academic and pedagogical content of the DER was deemed useful in terms of providing a sense of security and pedagogical efficiency; moreover, it acted as a flexible and accessible resource that enabled educators to acquire information rapidly and at their own pace. The usefulness of the DER was especially evident for the part-time and novice educators. All respondents agreed that DigiViS provided them with a highly needed foundation and supplement to support and transform their educational role in a way that would positively impact student learning.

Consistent with previous research [4, 18], our findings indicate that novice and part-time nurse educators need sufficient orientation, training, and support to enhance their role as educators in clinical nursing education, in particular due to their limited educational experience and lack of continuity with supervision and assessment in higher nursing education [4]. Research stresses that student learning may be hindered by part-time and novice educators, who have limited knowledge of the curriculum and are unsure about effective teaching and learning strategies and how to effectively assess clinical competence [28]. Nonetheless, preparation and orientation programs offered to novice or part-time nurse educators prior to clinical education are inadequate, associated with variability, and lack a systematic approach [11, 29].

There is, in fact, no consensus concerning best practices for the orientation of part-time educators due to a dearth of research in this area [14, 28]. This is surprising given the fact that there is a growing concern about part-time hired faculty in nursing education [11, 16]. Nurses who transition into an educator role without receiving appropriate formal preparation or support often experience role ambiguity and high levels of stress [4]. Consistent with previous research [14], the novice educators and clinical nurses hired part-time to act as nurse educators in our study reported feeling overwhelmed by the academic role and the content to be absorbed prior to placement and thus valued the flexibility provided in the DER.

By providing the educators with academic information about the students’ educational context, description of their learning outcomes, and nursing competencies to be pursued and assessed, the DER helped and motivated the educators to link theory and practice in a more efficient manner and promoted the use of reflective thinking. Corresponding with existing literature [13, 18], our findings suggest that online orientation components can enhance the content provided through in-person encounters as they allow for flexibility and can be completed at one’s own pace. Hence, the findings of the current study indicate that nurse education institutions need to develop and adopt more innovative strategies in formal orientation and preparation programs, including digital resources, to support the educator’s role and enhance student nurses’ learning and professional development in clinical nursing education. Receiving appropriate formal orientation may, moreover, contribute to increased satisfaction and have a positive impact on retention: part-time clinical nurses may consider nursing education a viable career option, which is paramount given the shortage of nurse educators [18].

Formal educational preparation should include pedagogical training, curriculum development, student assessment techniques, and management of students throughout their learning process [4]. However, the literature stresses that solely online orientation and training programs are not recommended due to the lack of face-to-face interactions [30]. Mentorship support from more experienced educators is another strategy used to assist novice educators in the development of required nurse educators’ competencies [4]. Based on study findings, we argue, in line with others [30], that further research is needed on innovative strategies and the effectiveness and combination of various forms of orientations for part-time and novice educators to ensure evidence-based practices in this area. Future research is also required to systematize knowledge about the impact of DERs on orientation and training, as well as motivation and facilitators for, and barriers to, their use to enhance quality and strengthen the nurse educator’s role in clinical nursing education.

Another concern raised by the literature concerning part-time educators is the lack of commitment to educational institutions as teaching and supervision represent a secondary job for these educators [31]. We did not record the participants’ level of participation in the orientation and follow-up meetings offered at the enrolled HEI, which represents a limitation of this study. However, based on study findings, we argue that requirements and attendance-promoting strategies to enhance novice and part-time nurse educators’ motivation to participate in orientation, learning, and development programs should be considered by educational nursing institutions. The participants in our study all expressed enthusiasm about the DER, and study findings indicate that access to DigiViS prior to and during the placement period increased the participants’ motivation as educators.

A recent literature review on the expectations of the nurse educator’s role in clinical placement from the perspective of the students, RN preceptors, and educators identified four different roles: pedagogical, academic, relational, and innovative [32]. This finding emphasizes the multiplicity and complexity of roles and responsibilities that the nurse educator is expected to undertake in clinical education and that fall to educational institutions. Participants in our study consistently expressed a positive attitude toward DigiViS and emphasized the potential for further development to address the complexity of the educational role in clinical nursing education. Finally, the use of DERs to support orientation, training, and development may also contribute to enhancing digital competence and support nurse educators’ engagement in technology-based learning, which is essential in the digital age [33].

### Study limitations

The findings of this study were based on a relatively small sample from a single educational institution in Norway. This raises the question about transferability of our research. However, we do believe that reports from use of innovative interventions such as DER and its following evaluation results are of significance for an international context, particularly for nursing education programs that employ the preceptorship model in clinical nursing education. Potential research biases should be acknowledged, because the data collection was conducted by insider researchers [34, 35] connected with the same educational institution as the participants enrolled in the study. We attempted to control for research biases by applying continuous self-reflection [34], ensuring that ethical practices were maintained and that the collected data was representing the participants authentic views in a satisfactory manner. In addition, we applied triangulation during the data analysis process [36] whereas all authors contributed to analytical integrity by validating the interpretations of the dataset, including the dissemination of the findings, by ensuring inner coherence and credibility. We also acknowledge that because of the Covid-19 pandemic the interviews were performed electronically, which limited the researchers’ ability to read social cues. Moreover, the participants may have evaluated DigiViS more positively during Covid-19, when there were no physical meetings for training and support due to pandemic measures. 

## Conclusions

Our findings suggest that supportive interventions such as DERs can represent innovative, efficient, and useful supplementary strategies to support and enhance the nurse educator’s role in clinical nursing education. The DER evaluated in this study provided useful academic and pedagogical support in a flexible and efficient manner, including knowledge of curriculum development and the student’s educational context, appropriate teaching, and learning strategies to facilitate and optimize student learning. In line with previous research, our findings call attention to the need for more formal and organized preparation and orientation to provide part-time and novice educators with sufficient training and support to ensure their (beginning competency) effectiveness, learning, and development as educators in clinical nursing education. Moreover, requirements and attendance-promoting strategies to participate in orientation, learning, and development programs should be addressed by educational nursing institutions, as this is essential for sustainable high-quality clinical education. The relative novelty of the use of DERs to support nurse educators in clinical nursing education means that there are still knowledge gaps to be explored. Future research is required to systematize knowledge about the impact of DERs on orientation and training, as well as motivation and facilitators for, and barriers to, their use to enhance quality and strengthen the nurse educator’s role in clinical nursing education. Nurse education plays a significant role in shaping the future of nursing, and nurse educators have an important role in promoting high-quality education, including teaching in clinical nursing education.

### Supplementary Information

**Addditional file 1: Supplementary File 1.** Standards for Reporting Qualitative Research guidelines.

## Data Availability

The dataset used and analyzed in the current study is available from the corresponding author upon reasonable request.

## Abbreviations

RN: Registered nurse
HEI: Higher educational institution
DER: Digital educational resource
FGA: Focus group A
FGB: Focus group B
#P: Participant
